# Atypical Presentation of Chikungunya in a 64-Year-Old Man: A Case Report From the UAE

**DOI:** 10.7759/cureus.114696

**Published:** 2026-08-17

**Authors:** Tasnim Moustafa, Mahnoor Dilawar, Farah Osama, Sarah I Zahid

**Affiliations:** 1 Internal Medicine, Gulf Medical University, Ajman, ARE; 2 Internal Medicine, College of Medicine, Gulf Medical University, Ajman, ARE

**Keywords:** arboviral disease, atypical presentation, case report, chikungunya, imported infection, older adult, united arab emirates

## Abstract

A 64-year-old man presented with a five-day history of high fever, diffuse joint pain, and generalized weakness following recent travel from Sri Lanka. On arrival, he appeared acutely ill, with significant pain-related immobility but no rash. Laboratory evaluation revealed mild leukopenia, lymphopenia, and elevated inflammatory markers, while initial serology was inconclusive. During hospitalization, he developed persistent fatigue, transient hypotensive episodes, and marked difficulty ambulating, necessitating close monitoring. Management included intravenous fluids, nonsteroidal anti-inflammatory drug (NSAID)-based analgesia, rest, and observation for complications such as persistent joint symptoms or cardiovascular instability. Over seven days, his fever resolved, blood pressure stabilized, and mobility gradually improved, allowing discharge in stable condition.

This case highlights the importance of recognizing atypical presentations in older adults with fever and severe joint pain, particularly in patients with recent travel from regions with ongoing arboviral transmission. Close monitoring and supportive care facilitated recovery, and reporting such cases can aid in the timely recognition and management of similar presentations in nonendemic settings.

## Introduction

Chikungunya is a mosquito-borne viral infection caused by an alphavirus transmitted primarily by Aedes mosquitoes [1]. The infection typically presents with sudden onset of fever, intense polyarthralgia, and rash, and most patients recover fully within a few weeks [2]. Although these features are characteristic, older adults may present with less typical manifestations, including prolonged fatigue, transient hypotension, or unusual musculoskeletal involvement, which may complicate recognition and diagnosis [3]. Global travel has facilitated the importation of chikungunya into non-endemic areas, underscoring the need for high clinical vigilance among practitioners in such regions [4]. In returning travelers, the clinical presentation may overlap with other mosquito-borne infections, and the absence of classical features such as rash may further contribute to diagnostic uncertainty [3]. Recognizing and reporting atypical cases is important for improving clinical awareness, supporting timely diagnosis, and optimizing patient care [5].

This report describes a 64-year-old man in the United Arab Emirates whose presentation differed from the typical clinical picture following recent travel from Sri Lanka [6]. The absence of rash, severe polyarthralgia causing marked functional limitation, and transient hypotensive episodes highlight the variability of chikungunya presentation in older adults. Reporting this case contributes to the recognition of atypical and travel-associated chikungunya presentations and emphasizes the importance of considering imported arboviral infection in patients presenting with fever and severe joint symptoms [6].

## Case presentation

A 64-year-old man presented to the emergency department with a five-day history of high-grade fever, severe diffuse joint pain, and pronounced generalized weakness, which had progressively limited his mobility and daily activities [4]. He reported that the joint pain was so intense that it interfered with basic tasks such as walking, dressing, and eating, with the wrists, knees, and ankles being most affected [1]. The patient also experienced intermittent chills, a mild frontal headache, and profound malaise but denied nausea, vomiting, diarrhea, cough, or shortness of breath. There was no history of recent medication use, noticeable insect bites, or contact with individuals with similar symptoms. His medical history was notable only for well-controlled hypertension managed with a single oral antihypertensive agent, with no history of diabetes mellitus, autoimmune disorders, or chronic joint diseases [4]. Importantly, he had recently returned from a three-week visit to Sri Lanka, a region with active outbreaks of mosquito-borne illnesses, including dengue and chikungunya, raising suspicion for an imported arboviral infection [2].

On examination, the patient appeared visibly ill, fatigued, and in moderate distress due to pain, with limited ability to ambulate independently. He had a slightly pale complexion and a cautious gait, with significant joint stiffness. Physical examination revealed mild tenderness and swelling in multiple joints, particularly the wrists and knees, without deformities, nodules, or erythema. No skin rash, petechiae, or mucosal lesions were noted. Cardiovascular and respiratory examinations were unremarkable, and the abdomen was soft and nontender. Vital signs showed intermittent hypotensive episodes (blood pressure ranging from 95/60 to 110/70 mmHg), a temperature of 38.2°C, and stable pulse and respiratory rates. Neurological assessment revealed intact cranial nerves and normal sensorimotor function, although mobility was limited by pain [4].

Initial laboratory investigations revealed mild leukopenia and lymphopenia, along with elevated erythrocyte sedimentation rate (ESR) and C-reactive protein (CRP) levels, while liver and renal function tests remained within normal limits. Electrolyte levels were normal, and urinalysis was unremarkable. Malaria and dengue tests were negative. Subsequent chikungunya testing showed positive IgM and a positive PCR result, confirming the diagnosis. The patient’s detailed laboratory results, including dengue NS1 antigen, dengue IgM/IgG, chikungunya IgM, and PCR results, are summarized in Table 1. Blood cultures were sterile. He was admitted for observation, symptomatic management, and further evaluation [4]. During hospitalization, his condition was closely monitored for hemodynamic stability, as he experienced intermittent mild dizziness associated with low blood pressure. Over the subsequent days, his fever persisted intermittently, and joint pain remained debilitating, requiring acetaminophen and nonsteroidal anti-inflammatory drugs for adequate pain relief [4].

**Table 1:** Laboratory Findings of the Patient on Admission Laboratory investigations demonstrated mild leukopenia and thrombocytopenia, with elevated erythrocyte sedimentation rate (ESR) and C-reactive protein (CRP), indicating systemic inflammation. Liver and renal function tests, electrolytes, and urinalysis were within normal limits. Dengue testing was negative, and Chikungunya serology and PCR confirmed infection. WBC: white blood cell count; ESR: erythrocyte sedimentation rate; CRP: C-reactive protein; AST: aspartate aminotransferase; ALT: alanine aminotransferase; ALP: alkaline phosphatase; NS1 Ag: dengue nonstructural protein 1 antigen; IgM: immunoglobulin M; IgG: immunoglobulin G; PCR: polymerase chain reaction; Chikungunya IgM: chikungunya virus-specific immunoglobulin M.

Laboratory Test	Patient Value	Reference Range
Hemoglobin	14.2 g/dL	13.0-17.0 g/dL
Hematocrit	42%	40-50%
WBC count	3800/µL	4000-11,000/µL
Neutrophils	60%	40-70%
Lymphocytes	30%	20-45%
Platelets	110,000/µL	150,000-450,000/µL
ESR	38 mm/hr	<20 mm/hr
CRP	18 mg/L	<5 mg/L
AST	32 U/L	10-40 U/L
ALT	30 U/L	7-56 U/L
ALP	90 U/L	44-147 U/L
Creatinine	1.0 mg/dL	0.6-1.3 mg/dL
Urea	16 mg/dL	7-20 mg/dL
Sodium	138 mmol/L	135-145 mmol/L
Potassium	4.1 mmol/L	3.5-5.0 mmol/L
Dengue NS1 Ag	Negative	Negative
Dengue IgM/IgG	Negative	Negative
Chikungunya IgM	Positive	Negative
Chikungunya PCR	Detected	Not detected

Supportive management included intravenous hydration, careful monitoring of fluid balance, and gradual mobilization as tolerated [4]. The patient was counseled on rest, joint protection during the acute phase, and maintaining adequate hydration. A physical therapy consultation was obtained to assist with joint mobility and prevent stiffness. Over the subsequent seven days, his fever resolved completely, appetite improved, and blood pressure stabilized without further hypotensive episodes. Laboratory parameters progressively normalized, and the positive chikungunya IgM and PCR results, together with the compatible clinical presentation and recent travel history, established the diagnosis of chikungunya infection [4,5]. At discharge, he was afebrile, able to ambulate independently, and reported marked improvement in joint pain, although mild morning stiffness persisted [5].

He was discharged with recommendations to continue oral hydration, engage in light physical activity, and use analgesics as needed for residual discomfort [4]. Follow-up emphasized monitoring for delayed or chronic arthralgia, which can occur in older adults recovering from chikungunya infection, and ongoing evaluation by his primary care physician. This case highlights the importance of maintaining a high index of suspicion for arboviral infections in returning travelers, particularly in nonendemic regions [3]. It also underscores the potential for atypical presentations and prolonged recovery in older patients, demonstrating the value of early supportive care, multidisciplinary management, and patient education in achieving favorable outcomes [6,7].

## Discussion

Chikungunya virus infection is increasingly recognized as a major public health concern due to its potential for rapid geographic spread and variable clinical presentation, particularly in older adults [3]. While fever and severe polyarthralgia are characteristic features of chikungunya infection, rash is also commonly reported [4]. In this case, leukopenia, thrombocytopenia, and elevated ESR and CRP levels were consistent with an acute infectious and inflammatory process. These findings may overlap with those of other arboviral infections, particularly dengue; however, negative dengue testing and positive chikungunya PCR supported the diagnosis [4]. Although the main clinical features were characteristic of chikungunya infection, the absence of rash, severe functional impairment due to joint pain, and transient hypotension made this case noteworthy. The patient’s age and hypertension were relevant clinical characteristics, but their contribution to the presentation cannot be established from a single case [6].

Supportive management remains the cornerstone of chikungunya treatment, as no specific antiviral therapy is currently available [1]. This includes adequate hydration, pain and fever control, and monitoring for complications such as hemodynamic instability and persistent joint pain [4,5]. In this case, the patient experienced transient hypotension, with a lowest documented blood pressure of 95/60 mmHg, accompanied by mild dizziness. The hypotension resolved with supportive management, without evidence of persistent hemodynamic instability or organ dysfunction. Although the exact cause could not be established, it may have been related to the acute febrile illness. Severe joint pain also caused significant functional limitation, requiring analgesia and physical therapy. The patient subsequently improved over one week, supporting the value of timely supportive care [4,7].

The patient’s favorable recovery underscores the value of early recognition, vigilant monitoring, and a holistic approach to care [1,4]. Reporting atypical and travel-related cases is essential to help clinicians worldwide recognize the diverse manifestations of chikungunya and respond appropriately [2]. Documentation of cases in nonendemic regions such as the United Arab Emirates also highlights the risk of imported infections and underscores the need for continued surveillance, clinician education, and effective patient counseling to prevent misdiagnosis or delayed intervention [6]. Furthermore, this case illustrates the broader principle that factors such as age, comorbidities, and immune response can significantly influence the clinical course of viral infections [5]. Sharing such findings through case reports contributes to expanding the current understanding of disease variability, improving diagnostic accuracy, and refining management strategies. Overall, these reports support evidence-based practice and strengthen preparedness for emerging infectious diseases across diverse populations [3,7].

## Conclusions

This case highlights the importance of considering chikungunya infection in returning travelers presenting with fever and severe polyarthralgia, even in the absence of rash. Although the patient’s main clinical features were characteristic of chikungunya, the absence of rash and transient hypotension were noteworthy. Clinicians in nonendemic regions should maintain a high index of suspicion for imported arboviral infections and use appropriate diagnostic testing to facilitate timely diagnosis and supportive management.
